# Prevalence and associated factors of dyslipidaemia among university workers in Southeast Nigeria: a cross-sectional study

**DOI:** 10.1186/s13690-021-00600-9

**Published:** 2021-05-13

**Authors:** Adaobi M. Okafor, Elizabeth K. Ngwu, Rufina N.B. Ayogu

**Affiliations:** Department of Nutrition and Dietetics, University of Nigeria, Nsukka, Nigeria

**Keywords:** Hypercholesterolemia, high low density lipoprotein, hypertriglyceridemia, low high density lipoprotein, atherogenic index, university staff, Nigeria

## Abstract

**Background:**

The job one does for a living may increase ones propensity to cardiovascular diseases due to many associated risk factors. University staff may be at high risk of dyslipidaemia, a major cardiovascular disease risk factor. This study assessed prevalence of dyslipidaemia and its associated factors among the staff of University of Nigeria, Nsukka campus, Nigeria.

**Methods:**

A cross-sectional survey of 386 workers selected through a 4-stage sampling technique was conducted. Data were obtained through questionnaire and lipid profile determination. Bivariate analysis using Cochran and Mantel-Haenszel test was used to determine associations between dyslipidaemia and selected variables. Odds ratios and significance at *p* < 0.05 were reported.

**Results:**

Respondents who were 46 years and above accounted for 51.3 % while 95.3 % had tertiary education. Administrative/technical staff were 76.4 % while academic staff were only 23.6 %; 73.8 % were senior staff and 26.2 % were junior staff. More than half (60.4 %) consumed alcohol above recommendation. Lipid biomarkers of the workers were not sex dependent (*p* > 0.05). Few (23.4 and 6.5 %) of the respondents had borderline high and high total cholesterol values, respectively. Whereas none (0.0 %) had low high density lipoprotein cholesterol (HDL-c), borderline low values were observed among 1.3 %. High low density lipoprotein cholesterol (LDL-c) affected 1.3 %. Triglyceride was high among 3.9 %; 20.8 % had high atherogenic index of plasma (AIP) and 2.6 % had impaired fasting blood glucose (IFBG). Dyslipidaemia had a prevalence of 54.5 % with female dominance. Hypercholesterolemia with high LDL-c was the commonest combined dyslipidaemia observed (7.8 %). Dyslipidaemia was dependent on hypercholesterolemia (OR = 0.352, 95 % C.I.=0.245–0.505), high LDL-c (OR = 0.462, 95 % C.I.=0.355-0.600) and hypertriglyceridemia (OR = 0.462, 95 % C.I.=0.355-0.600). Alcohol intake above normal was associated with almost 6 times higher risk of dyslipidaemia (OR = 5.625, 95 % C.I.=1.062–29.799).

**Conclusions:**

Dyslipidaemia is a problem among the workers with hypercholesterolemia in combination with high LDL-c and hypertriglyceridemia compounding the problem. Nutrition education and physical activity are advocated to prevent cardiovascular events among the university staff.

## Background

Dyslipidaemia is an asymptomatic key independent modifiable cardiovascular disease (CVD) risk factor described as a group of metabolic disorders characterized by any or a combination of raised total cholesterol (TC), raised low-density lipoprotein cholesterol (LDL-c), raised triglycerides (TG) and low high-density lipoprotein cholesterol (HDL-c) that often leads to a persistent increase in the plasma concentration of cholesterol and triglycerides [1]. According to WHO [2], raised blood cholesterol increases the risks of heart diseases and stroke contributing to a third of ischaemic heart disease globally. Blood levels of low-density lipoprotein cholesterol (LDL-c) significantly predict incident atherosclerotic cardiovascular disease and LDL-c-lowering therapy has been repeatedly demonstrated in many populations to reduce CVD risk [3]. CVDs have been described as a major cause of death globally with more people dying annually from CVDs than from any other cause [4].

The nature of work and work environment have been linked with dyslipidaemia. The many aspects of work which may be related to this are unhealthy diets, physical inactivity, mental and physical work related stress. In a study on the relationship of job stress and dyslipidaemia, Catalina-Romero et al. [5] showed that job stress was associated with dyslipidaemia even after adjusting for confounding variables. Dyslipidaemia has also been reported among workers in various occupational categories. Work in rail road industry was a risk factor associated with high levels of low density lipoprotein especially among shift workers [6]. Kang et al. [7] also reported that work in companies and related job mental stress especially in the background of decision-making was related to high amount of blood cholesterol and triglycerides among the workers. Employed workers spend a quarter of their lives at work and the pressure and demands of work may affect their eating habits, lifestyle and activity patterns with serious harm to the overall health of the workers.

Work at the University is not exempt from physical and mental stress besides being majorly sedentary hence its staff may be at risk of dyslipidaemia and therefore CVDs. Sedentary behaviours are associated with adverse metabolic profiles [8] commonly seen in dyslipidaemia. This coupled with limited opportunity for other forms of activity and predisposition to a notable level of stress associated with work and family obligations may further exacerbate the susceptibility of workers in the university to dyslipidaemia. Sedentary lifestyle and physical inactivity either individually or in combination are known precursors of atherogenic risk [8], a useful predictive parameter for guiding prompt and timely interventions.

Evaluating the prevalence of dyslipidaemia and atherogenic risk of university staff is an important step in the planning of health promotion programmes for prevention of dyslipidaemia and its negative clinical effects [9]. Since little is known about the prevalence and factors associated with dyslipidaemia among University staff in Nigeria, it was, therefore, important to determine the prevalence of dyslipidaemia and factors associated with it among them. This will provide data vital to intervention planning, implementation and evaluation.

## Methods

### Area of study

The study was conducted within the months of August and October at Nsukka campus of the University of Nigeria. The university has nine faculties with a total of about 4,526 academic and administrative/technical staff.

#### Study design

A cross-sectional survey design was adopted for this study.

#### Sample size and sampling technique

Sample size for the study was 386 university staff of both sexes obtained through a single population formula (Yamane’s formula) based on 95 % precision. 20 % of this formed the subsample on which biochemical tests were conducted. The respondents were selected from all faculties of the University of Nigeria, Nsukka campus (the main campus of the university) through multistage random sampling. All nine faculties in the University of Nigeria, Nsukka campus were selected and three departments from each faculty were also selected by simple random sampling technique to yield a total of 27 departments. Proportionate sampling was used to obtain the number of respondents to be selected from each department. Final selection of the participants was through simple random sampling by balloting without replacement. Respondents (77) for biochemical examination were selected from the sample through simple random sampling. All staff healthy enough to be on duty were included in the study.

#### Ethical approval and informed consent

 Ethical approval (NHREC/05/01/2008B) was obtained from the Health Research Ethics Committee of the University of Nigeria Teaching Hospital, Ituku-Ozalla, Enugu. Informed consent was obtained from the respondents in writing after detailed explanation of the study protocol prior to recruitment into the study. Confidentiality was assured and maintained.

#### Methods of data collection

##### Questionnaire

Questionnaire used for the study was constructed to elicit socio-economic and lifestyle characteristics of the respondents. Face and content validation of the questionnaire was done by five Lecturers in the Department of Nutrition and Dietetics, University of Nigeria, Nsukka. Corrections were effected and the project supervisor approved the final copy of the questionnaire for distribution. It was interviewee administered with 100 % response rate.

*Lipid profile measurement*: Five millilitres of blood was obtained by a laboratory scientist from each respondent after 10 h post-absorptive fast. The blood was transferred into well-labelled plain specimen bottles and allowed to stand for 30 min at room temperature for complete clotting and clot retraction. Samples were centrifuged at 3500 revolution/minute for 15 min for extraction of clear serum. The serum was used for analysis of total cholesterol, high-density lipoprotein cholesterol (HDL-c), low-density lipoprotein cholesterol (LDL-c) and triglyceride (TG) using Randox kit. Dyslipidaemia was classified according to the Executive summary of the third report of the National Cholesterol Education Programme/Adult Treatment Panel III (NCEP/ATP III) [10] as the presence of any of the following: hypercholesterolemia (≥ 200 mg/dl), high LDL-c (≥ 130 mg/dl), low HDL-c (≤ 40 mg/dl in men; ≤50 mg/dl in women) and hypertriglyceridemia (≥ 150 mg/dl). Atherogenic index of plasma (AIP) was calculated and categorised as low risk (< 0.11), intermediate risk (0.11–0.21) and high risk (> 0.21) [11].

##### Fasting blood glucose measurement

A 3-day fasting blood glucose measurements (10 h of post-absorptive or over-night fast) were taken using an Accu-Chek glucometer; the mean was used in analysis. Categories of fasting blood glucose used were low blood glucose (< 65 mg/dl); normal blood glucose (65 - <110 mg/dl); impaired fasting blood glucose (110–125 mg/dl) and diabetes mellitus (≥ 126 mg/dl) [12].

#### Statistical Analysis

 Data obtained were analysed using the computer programme, Statistical Product and Service Solution, version 18. Data obtained were presented as frequencies, percentages, means and standard deviations. T-test and Chi-square analysis were used to determine relationships among variables. Crude odds ratios generated through Cochran’s and Mantel-Haenszel statistics were reported. Significance was accepted at *p* < 0.05.

## Results

Table 1 shows the general characteristics of the respondents. Majority (51.3 %) of the respondents were aged 46 years and above with more males (65.0 %). More females (97.3 %) than males (94.2 %) had tertiary education. Majority were administrative/ technical staff (76.4 %) and senior staff (76.0 %). Monthly basic income was above ₦50, 000 (138.7 USD) among 55.2 % of the respondents. Consumption of alcohol above normal was observed in more females (61.5 %) than males (59.6 %).

**Table 1:** General characteristics of the respondents

Variables	Male	Female	Total
	**N (%)**	**N (%)**	**N (%)**
**Age (years)**			
18–45	84 (35.0)	104 (71.2)	188 (48.7)
≥ 46	156 (65.0)	42 (28.8)	198 (51.3)
**Total**	**240 (100.0)**	**146 (100.0)**	**386 (100.0)**
**Educational level**			
Secondary education	14 (5.8)	4 (2.7)	18 (4.7)
Tertiary education	226 (94.2)	142 (97.3)	368 (95.3)
**Total**	**240 (100.0)**	**146 (100.0)**	**386 (100.0)**
**Staff category**			
Academic	45 (18.8)	46 (31.5)	91 (23.6)
Administrative/ Technical	195 (81.2)	100 (68.5)	295 (76.4)
**Total**	**240 (100.0)**	**146 (100.0)**	**386 (100.0)**
**Cadre**			
Junior	66 (27.5)	35 (24.0)	101 (26.2)
Senior	174 (72.5)	110 (76.0)	284 (73.8)
**Total**	**240 (100.0)**	**146 (100.0)**	**386 (100.0)**
**Total**	**240 (100.0)**	**146 (100.0)**	**386 (100.0)**
**Monthly basic income (₦)**			
≤₦50, 000 (≤ 138.7 USD)	113 (47.1)	60 (41.1)	173 (44.8)
>₦50, 000 (> 138.7 USD)	127 (52.9)	86 (58.9)	213 (55.2)
**Total**	**240 (100.0)**	**146 (100.0)**	**386 (100.0)**
**Alcohol intake**			
Normal	69 (40.4)	40 (38.5)	109 (39.6)
Above normal	102 (59.6)	64 (61.5)	166 (60.4)
**Total**	**171 (100.0)**	**104 (100.0)**	**275 (100.0)**

Normal alcohol intake/day: ≤ 2units (males), ≤ 1 unit (females)

Mean biochemical parameters of the respondents by sex are presented in Table 2. Females had higher values of total cholesterol (189.9±37.4 mg/dl) and high density lipoprotein (82.6±10.4 mg/dl) but lower atherogenic index of plasma (AIP) (-0.3±0.2) than males. Values of low density lipoprotein (103.1±27.4 mg/dl), triglyceride (117.7±48.7 mg/dl) and fasting blood glucose (92.5±7.8 mg/dl) were higher among males. These differences were not significant (*P* > 0.05).

**Table 2:** Mean biochemical parameters of the respondents by sex

Variables	Male	Female	Group mean	t-value, *p*-value
**Lipid profile (mg/dl)**				
Total cholesterol	178.3 ± 23.6	189.9 ± 37.4	186.1 ± 34.0	1.391, 0.168
High-density lipoprotein	80.3 ± 12.4	82.6 ± 10.4	81.8 ± 11.2	0.866, 0.594
Low-density lipoprotein	103.1 ± 27.4	99.6 ± 25.9	100.8 ± 26.3	0.535, 0.594
Triglyceride	117.7 ± 48.7	99.1 ± 38.9	104.4 ± 43.4	1.826, 0.720
**AIP**	-0.2 ± 0.2	-0.3 ± 0.2	-0.3 ± 0.2	1.677, 0.099
**FBG (mg/dL)**	92.5 ± 7.8	91.8 ± 8.5	92.0 ± 8.2	0.356, 0.723

^*AIP* Atherogenic index of plasma; *FBG  *Fasting blood glucose Values are mean±SD *p<0.05^

Table 3 displays the lipid profile status of the respondents by sex. Borderline high (23.4 %) and high (6.5 %) serum total cholesterol were observed among the respondents. Majority (98.7 %) had desirable serum HDL-c. Only 14.3 and 1.3 % of the respondents had borderline high and high serum LDL-c, respectively. A few had borderline high (11.7 %) and high (3.9 %) serum triglyceride. With atherogenic index of plasma, majority (79.2 %) had low CVD risk. Impaired fasting blood glucose existed in 2.6 % of the respondents. Diabetes was not observed. These observed differences were not sex dependent (*p* > 0.05).

**Table 3:** Lipid profile status of the respondents by sex

	Male	Female	Total	
**Variables**	**N(%)**	**N(%)**	**N(%)**	***p* value**^**a**^
**Total cholesterol (mg/dl)**				
Desirable (< 200)	20(80.0)	34(65.4)	54(70.1)	
Borderline high (200–239)	5(20.0)	13(25.0)	18(23.4)	
High (≥ 240)	0(0.0)	5(9.6)	5(6.5)	0.212
**Total**	**25(100.0)**	**52(100.0)**	**77(100.0)**	
**HDL-c (mg/dl)**				
Low (< 40)	0(0.0)	0(0.0)	0(0.0)	
Borderline (40–59)	0(0.0)	1(1.9)	1(1.3)	
Desirable (≥ 60)	25(100.0)	51(98.1)	76(98.7)	
**Total**	**25(100.0)**	**52(100.0)**	**77(100.0)**	1.000
**LDL-c (mg/dl)**				
Optimal (< 100)	10(40.0)	24(46.2)	34(44.2)	
Above optimal (100–129)	12(48.0)	19(36.5)	31(40.3)	
Borderline high (130–159)	2(8.0)	9(17.3)	11(14.3)	
High (160–189)	1(4.0)	0(0.0)	1(1.3)	0.284
**Total**	**25(100.0)**	**52(100.0)**	**77(100.0)**	
**Triglyceride (mg/dl)**				
Normal (< 150)	18(72.0)	47(90.4)	65(84.4)	
Borderline high (150–199)	5(20.0)	4(7.7)	9(11.7)	
High (200–499)	2(8.0)	1(1.9)	3(3.9)	0.302
**Total**	**25(100.0)**	**52(100.0)**	**77(100.0)**	
**Atherogenic index of plasma**				
Low cardiovascular risk (< 0.11)	21 (84.0)	40 (76.9)	61 (79.2)	
Intermediate cardiovascular risk (0.11–0.21)	4 (16.0)	12(23.1)	16 (20.8)	
High cardiovascular risk (> 0.21)	0 (0.0)	0 (0.0)	0 (0.0)	0.485
**Total**	**25 (100.0)**	**52(100.0)**	**77(100.0)**	
**Fasting blood glucose (FBG)**				
Normal (65-<110 mg/dl)	24(96.0)	51(98.1)	75(97.4)	
Impaired FBG (110-125 mg/dl)	1(4.0)	1(1.9)	2(2.6)	
Diabetes mellitus (≥ 126 mg/dl)	0(0.0)	0(0.0)	0(0.0)	0.547
**Total**	**25(100.0)**	**52(100.0)**	**77(100.0)**	

^a^Values were generated through chi square analysis *P < 0.05

*HDL-c* High density lipoprotein cholesterol *LDL-c* Low density lipoprotein cholesterol

**Fig. 1:**
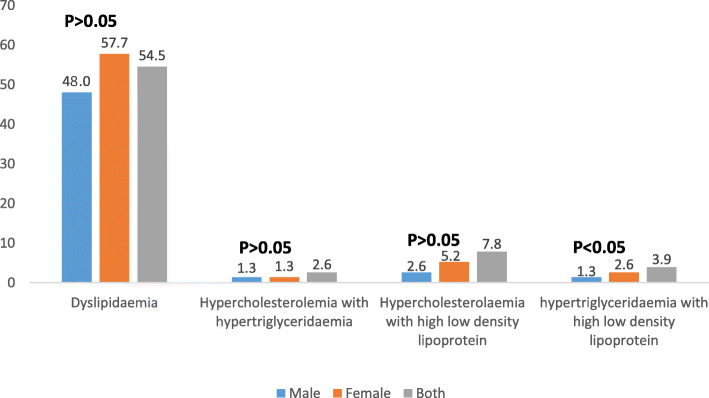
Prevalence of dyslipidaemia and combined dyslipidaemia by sex

Figure 1 illustrates the prevalence of dyslipidaemia and combined dyslipidaemia by sex. The prevalence of dyslipidaemia was higher among females (57.7 %) than males (48.0 %). Hypercholesterolemia with hypertriglyceridemia has the same prevalence for males and females (1.3 %). Prevalence of hypercholesterolemia with high low density lipoprotein was lower among females (2.6 %) than males (5.2 %). These differences were not significant (*p* > 0.05). Females (2.6 %) had significantly (*p* < 0.05) higher prevalence of hypertriglyceridemia with high low density lipoprotein than males (1.3 %).

Associated factors of dyslipidaemia are shown in Table 4. Prevalence of dyslipidaemia was dependent on hypercholesterolemia (OR = 0.352, 95 % C.I.=0.245–0.505, *p* < 0.001), high LDL-c (OR = 0.462, 95 % C.I.=0.355–0.600, *p* < 0.001) and hypertriglyceridemia (OR = 0.462, 95 % C.I.=0.355–0.600, *p* < 0.001). Participants who consumed alcoholic beverage above normal were almost 6 times at higher risk of dyslipidaemia (OR = 5.625, 95 % C.I.=1.062–29.799, *p* < 0.05).

**Table 4:** Associated factors of dyslipidaemia

	Dyslipidaemia				
**Variables**	**Present**	**Absent**	**OR**	**95 % C.I.**	***p* value**
**Hypercholesterolemia**					
Absent (< 200 mg/dl)	19 (45.2)	35 (100.0)	0.352	0.245–0.505	0.000***
Present (≥ 200 mg/dl)	23 (54.8)	0 (0.0)			
**Total**	**42 (100.0)**	**35 (100.0)**			
**High Low density lipoprotein cholesterol**					
Absent (< 130 mg/dl)	30 (71.4)	35 (100.0)	0.462	0.355–0.600	0.000***
Present (≥ 130 mg/dl)	12 (28.6)	0 (0.0)			
**Total**	**42 (100.0)**	**35 (100.0)**			
**Hypertriglyceridemia**					
Absent (< 150 mg/dl)	30 (71.4)	35 (100.0)	0.462	0.355–0.600	0.000***
Present (≥ 150 mg/dl)	12 (28.6)	0 (0.0)			
**Total**	**42 (100.0)**	**35 (100.0)**			
**Age (years)**					
18–45	2 (4.8)	3 (8.6)	0.533	0.084–3.387	0.654
≥ 46	40 (95.2)	32 (91.4)			
**Total**	**42 (100.0)**	**35 (100.0)**			
**Sex**					
Male	12 (28.6)	13 (37.1)	0.677	0.260–1.765	0.470
Female	30 (71.4)	22 (62.9)			
**Total**	**42 (100.0)**	**35 (100.0)**			
**Educational level**					
Secondary education	2 (4.8)	4 (11.4)	0.388	0.067–2.255	0.402
Tertiary education	40 (95.2)	31 (88.6)			
**Total**	**42 (100.0)**	**35 (100.0)**			
**Staff category**					
Academic	8 (19.0)	10 (28.6)	0.588	0.203–1.704	0.420
Administrative/Technical	34 (81.0)	25 (71.4)			
**Total**	**42 (100.0)**	**35 (100.0)**			
**Cadre**					
Junior	10 (23.8)	10 (28.6)	0.781	0.281–2.168	0.795
Senior	32 (76.2)	25 (71.4)			
**Total**	**42 (100.0)**	**35 (100.0)**			
**Basic monthly income (₦)**					
≤ 50, 000 (≤ 138.7 USD)	14 (33.3)	17 (48.6)	0.529	0.210–1.332	0.244
> 50,000 (> 138.7 USD)	28 (66.7)	18 (51.4)			
**Total**	**42 (100.0)**	**35 (100.0)**			
**Atherogenic index of plasma**					
Medium cardiovascular risk	13 (31.0)	3 (8.6)	0.357	0.125–1.019	0.378
Low cardiovascular risk	29 (69.0)	32 (91.4)			
**Total**	**42 (100.0)**	**35 (100.0)**			
**Alcohol** intake					
Normal intake	9 (36.0)	2 (9.1)	5.625	1.062–29.799	0.041*
Above normal intake	16 (64.0)	20 (90.9)			
**Total**	**25 (100.0)**	**22 (100.0)**			
**Fasting blood glucose**					
Normal	40 (95.2)	35 (100.0)	1.875	1.517–2.317	0.498
Impaired	2 (4.8)	0 (0.0)			
**Total**	**42 (100.0)**	**35 (100.0)**			

*P < 0.05 ***P < 0.001

## Discussion

Dyslipidaemia, which is one of the most important modifiable risk factors for many chronic non-communicable diseases (NCDs) especially CVDs was present among a high proportion of the respondents. The high prevalence observed in this study is worrisome though lower than earlier reports of 62.1 % among adults in northeast China [13], 78 % among primary health care employees in Jeddah, Saudi Arabia [14] and 59.74 % [15] among residents of Sao Paulo. This implies a propensity of majority of the workers to cardiovascular events with implications on morbidity and mortality among the workers if uncontrolled.

That hypercholesterolemia was the commonest dyslipidaemia observed among the respondents is contrary to the findings of Sani et al. [16] and Basheikh et al. [14] who reported low HDL-c as the commonest dyslipidaemia in Katsina (59.3 %) and Jeddah, Saudi Arabia (45.2 %). That more females relative to the males were affected by hypercholesterolemia may be consequent upon physical inactivity and dietary habits. Some authors have reported females as being less physically active than males [17, 18]. Besides, being in more contact with food through meal preparation and distribution, females may be predisposed to excessive food consumption. Excessive consumption of fast food and takeaway meals (associated with snacking in work places) which are energy-dense and has high total and saturated fats has also been associated with higher total and LDL cholesterol [19]. High total cholesterol is a known risk factor for developing atherosclerosis and other CVDs. Hypercholesterolemia alone is implicated in the process of atherogenesis and a curvilinear relationship between increased cholesterol levels and increased incidence of CVDs has been documented [20].

Desirable HDL-c which existed in almost all the respondents differed from 8.8 % reduced HDL-c reported earlier [21] and indicates that the respondents may to some extent be protected from CVDs. High HDL-c exerts a protective effect by decreasing the rate of entry of cholesterol into the cells and increasing the rate of cholesterol release from the cell by enhancing reverse cholesterol transport through scavenging excess cholesterol from peripheral tissues [22]. High HDL-c also inhibits the oxidation of LDL-c as well as the atherogenic effects of oxidized LDL-c by virtue of its antioxidant and anti-inflammatory properties. An earlier report showed that 1 mg increase in serum HDL-c correlates with 2 % reduction in the risk of coronary heart disease for men and 3 % for women [23].

High serum LDL-c which existed among the respondents has implications on their cardiovascular health because LDL-c is known to be pro-atherogenic. LDL-c modified by oxidation plays a key role in atherogenesis [24]. Oxidized LDL-c alters the structure and function of the endothelial cells, attracts monocytes and macrophages to the endothelium which then develops into the lipid laden foam cells of an atheromatous plaque. Depending on the type of LDL-c, the respondents in this study may be at risk of developing CVDs with time and being a work force, this may have implications on family and national economy and development.

Though not significant (*p* > 0.05), males had higher serum TG values relative to the females indicating high vulnerability of males to CVDs. This finding was a surprise since more females than males consumed alcohol above recommendation and heavy alcohol consumption has been associated with hypertriglyceridemia [25] in line with our observation that those who consumed above normal quantities of alcohol were 5.6 times at higher risk of dyslipidaemia. In a study among hypertensive men who consumed alcohol, Park and Kim [26] observed that the risk of high TG increased with increasing alcohol consumption making high plasma concentration of triglycerides both an independent and synergistic risk factor for CVDs.

The low cardiovascular risk based on atherogenic index of plasma (AIP) reported in this study could be attributed to high HDL-c because the value for AIP indicates a balance between the actual concentration of plasma triglycerides and HDL-c which predetermine the direction of cholesterol transport in the intravascular pool (i.e. the flux of newly produced cholesteryl esters by lecithin cholesterol acyltransferase) towards atherogenic LDL-c or beneficial HDL-c [27]. HDL-c exerts its cardio-protective effects mainly via its role in the reverse cholesterol transport pathway, by promoting the removal of cholesterol from peripheral cells and preventing atherogenesis [28]. Contrary to the findings of this study, higher prevalence of high (16.4 %) cardiovascular risk based on AIP has been reported [29]; high cardiovascular risk was also reported as 33.5 and 35.1 % among rural and urban dwellers in Abuja FCT [30]. Having more than one lipid abnormality has implication for increased risk of atherosclerosis and cardiovascular events. A study has shown that hypertriglyceridemia when associated with high LDL-c, significantly increases the risk of coronary heart diseases [15].

Older age has been shown to be a risk factor for dyslipidaemia [31] similar to the findings of this study where those aged ≥ 46 years had much higher prevalence of dyslipidaemia than their younger counterpart though this relationship was not significant (*p* > 0.05). In support, dyslipidaemia prevalence of 13.4 % was reported among ≥ 40 year-old employees of primary health care centre in Jeddah, Saudi Arabia compared to the < 40 year-olds [13]. Ageing causes increased sedentary life, reduced metabolism (cholesterol inclusive) and increased accumulation of body lipids with higher atherogenic risk.

## Limitation

This study centred only on university staff of one institution and does not reflect the situation in other institutions. It however has revealed the propensity of university staff to dyslipidaemia, an important CVD risk factor and the vulnerability of workers in similar work environment to CVD. Besides, the relatively small number used for biochemical analysis may have implication on the statistical power.

## Conclusions

Dyslipidaemia was prevalent among the respondents with hypercholesterolemia being the commonest dyslipidaemia. The commonest combined dyslipidaemia pattern seen among the respondents was hypercholesterolemia with high LDL-c. Atherogenic index of plasma revealed that the respondents were at low cardiovascular risk. Consumption of alcohol above normal quantity was associated with dyslipidaemia. Regular screening for dyslipidaemia is therefore recommended to enhance prompt identification, treatment and control, a vital step to prevention of CVDs in future. Nutrition and health education are also recommended to increase awareness and reduce the prevalence through lifestyle modification.

The authors did not receive funding for this research from any organization, governmental or nongovernmental.

## Data Availability

Dataset analysed during this study are available from the corresponding author on reasonable request.

## Abbreviations

CVDs: Cardiovascular diseases
HDL-c: High density lipoprotein
LDL-c: Low density lipoprotein
TC: Total cholesterol
TG: Triglyceride
AIP: Atherogenic index of plasma
NCD: Non-communicable diseases
OR: Odds ratio
C.I.: Confidence interval
